# Supplementary material for: Expression of EMP 1, 2, and 3 in Adrenal Cortical Neoplasm and Pheochromocytoma
Source: Int J Mol Sci. 2023 Aug 21;24(16):13016. doi: 10.3390/ijms241613016 (PMC10455306; doi:10.3390/ijms241613016)

Table S1. Source, clone, and dilution of antibodies

Antibody	Company	Clone	Dilution
EMP1	Abcam, Cambridge, UK	Polyclonal	1:100
EMP2	Abcam, Cambridge, UK	Polyclonal	1:50
EMP3	Abcam, Cambridge, UK	SW-5	1:100

Table S2. Basal characteristics of adrenal cortical neoplasm

Parameters	Total N=132 (%)	Adrenal cortical adenoma n=115 (%)	Adrenal cortical carcinoma n=17 (%)	p-value
Age (year, mean±SD)	47.5±145	48.4±12.2	41.0±25.1	**0.048**
Sex				0.107
Male	40 (30.3)	32 (27.8)	8 (47.1)	
Female	92 (69.7)	83 (72.2)	9 (52.9)	
Tumor size (cm, mean±SD)	3.6±3.7	2.5±1.3	10.9±5.8	**<0.001**
Fuhrman grade				**<0.001**
1 and 2	106 (80.3)	102 (88.7)	4 (23.5)	
3 and 4	26 (19.7)	13 (11.3)	13 (76.5)	
Mitosis				**<0.001**
≤ 5/50 HPFs	122 (92.4)	115 (100.0)	7 (41.2)	
> 5/50 HPFs	10 (7.6)	0 (0.0)	10 (58.8)	
Atypical mitosis				**<0.001**
Absent	121 (91.7)	114 (99.1)	7 (41.2)	
Present	11 (8.3)	1 (0.9)	10 (58.8)	
Clear cell proportion				**<0.001**
≥ 25%	96 (72.7)	95 (82.6)	1 (5.9)	
< 25%	36 (27.3)	20 (17.4)	16 (94.1)	
Diffuse architecture				**<0.001**
< 1/3	117 (88.6)	111 (96.5)	6 (35.3)	
≥ 1/3	15 (11.4)	4 (3.5)	11 (64.7)	
Necrosis				**<0.001**
Absent	113 (85.6)	113 (98.3)	0 (0.0)	
Present	19 (14.4)	2 (1.7)	17 (100.0)	
Venous invasion				**<0.001**
Absent	126 (95.5)	115 (100.0)	11 (64.7)	
Present	6 (4.5)	0 (0.0)	6 (35.3)	
Sinusoidal invasion				**<0.001**
Absent	126 (95.5)	115 (100.0)	11 (64.7)	
Present	6 (4.5)	0 (0.0)	6 (35.3)	
Capsular invasion				**<0.001**
Absent	117 (88.6)	111 (96.5)	6 (35.3)	
Present	15 (11.4)	4 (3.5)	11 (64.7)	
Weiss total score				**<0.001**
< 4	117 (88.6)	115 (100.0)	2 (11.8)*	
≥ 4	15 (11.4)	0 (0.0)	15 (88.2)	
Serum aldosterone level# (pg/mL, mean±SD)	262.3±269.8	272.0±278.0	160.7±128.5	0.239
Recurrence	3 (2.3)	0 (0.0)	3 (17.6)	**<0.001**
Distant metastasis	7 (5.3)	0 (0.0)	7 (41.2)	**<0.001**
Patient death	9 (6.8)	0 (0.0)	9 (52.9)	**<0.001**

SD, standard deviation
*Although the Weiss score was 4 or less, it was diagnosed as adrenal cortical carcinoma as metastases were present at the time of diagnosis.
Only 103 cases with serum aldosterone level data were included.

Table S3. Basal characteristics of pheochromocytoma

Parameters	Total, N=189 (%)
Age (year, mean±SD)	48.1±13.7
Sex	
Male	73 (38.6)
Female	116 (61.4)
Tumor size (cm, mean±SD)	5.0±3.4
Histologic pattern	
Zellballen	165 (87.3)
Non-Zellballen	24 (12.7)
Cellularity	
Low	11 (5.8)
Moderate	162 (85.7)
High	16 (8.5)
Comedo necrosis	
Absent	189 (100.0)
Present	0 (0.0)
Vascular or capsular invasion	
Absent	129 (68.3)
Present	60 (31.7)
Ki-67 labeling index (%)	
<1	139 (73.5)
1-3	38 (20.1)
>3	12 (6.3)
Catecholamine type*	
Non-norepinephrine type	154 (81.5)
Norepinephrine type	35 (18.5)
24 hour urine catecholamine level	

Epinephrine (μg/day, mean±SD)	99.9±198.3
Norepinephrine (μg/day, mean±SD)	386.1±1055.2
GAPP score	
0-2 (well-differentiated type)	138 (73.0)
3-6 (moderately differentiated type)	50 (26.5)
7-10 (poorly differentiated type)	1 (0.5)
Tumor recurrence	5 (2.6)
Distant metastasis	7 (3.7)
Patient death	11 (5.8)

* Only 138 cases with 24-hour urine catecholamine study data were included.

Table S4. Difference in IHC proportion score based on IHC intensity score in adrenal neoplasm

IHC proportion score (mean±SD)	Adrenal cortical neoplasm				Pheochromocytoma			
	IHC intensity score			p-value	IHC intensity score			p-value
	1	2	3		1	2	3	
EMP1	39.8±35.2	65.1±33.2	98.7±5.0	<0.001	74.1±31.5	84.6±23.9	98.7±3.5	0.011
EMP2	41.4±35.7	n/a	n/a	n/a	43.3±15.2	n/a	n/a	n/a
EMP3	52.3±38.7	66.9±35.6	92.0±10.9	0.045	84.6±25.8	90.1±16.5	100.0±0.0	0.111

Table S5. H-scores of EMP 1, 2, and 3 in adrenal cortical neoplasm

EMP type	Total N=132 (mean±SD)	Adrenal cortical adenoma, n=115 (mean±SD)	Adrenal cortical carcinoma, n=17 (mean±SD)	p-value
EMP1	91.6±101.0	73.6±89.6	213.5±91.0	**<0.001**
EMP2	2.2±12.0	2.5±12.8	0.0±0.0	0.423
EMP3	63.4±82.4	51.7±78.5	142.9±63.1	**<0.001**

Table S6. Serum aldosterone level according to EMP 1, 2, and 3 statuses in adrenal cortical neoplasm

EMP type	Serum aldosterone level (pg/mL, mean±SD)	p-value
EMP 1		**0.001**
Low	321.9±295.7	
High	146.5±158.7	
EMP 2		0.300
Low	268.5±273.7	
High	139.8±138.8	
EMP 3		**0.008**
Low	310.6±296.5	
High	159.9±162.9	

Table S7. H-scores of EMP 1, 2, and 3 in pheochromocytoma according to GAPP score

EMP type	Total<br>N=189 (mean±SD)	GAPP < 3<br>n=138 (mean±SD)	GAPP ≥ 3<br>n=51 (mean±SD)	p-value
EMP1	105.9±78.4	97.8±75.3	128.0±83.0	**0.018**
EMP2	0.6±5.6	0.9±6.6	0.0±0.0	0.311
EMP3	102.5±80.1	101.9±80.4	104.1±80.3	0.870

GAPP, grading system for adrenal pheochromocytoma and paraganglioma

Table S8. 24-hour urine catecholamine level according to EMP 1, 2, and 3 statuses in pheochromocytoma

EMP type	Epinephrine (μg/day, mean±SD)	p-value	Norepinephrine (μg/day, mean±SD)	p-value
EMP 1		0.633		0.354
Low	105.7±194.3		325.9±680.9	
High	88.6±207.5		502.4±1545.4	
EMP 2		0.321		0.718
Low	97.4±194.9		390.9±1066.2	
High	212.7±358.1		167.2±178.0	
EMP 3		0.731		0.504
Low	104.4±213.8		339.1±670.4	
High	92.4±171.3		463.6±1494.0	

Figure S1. Immunohistochemical stain for EMP 1, EMP2 and EMP3 in normal adrenal gland. EMP 1, EMP2 and EMP3 show membranous and/or cytoplasmic expression in adrenal cortical and adrenal medulla cells (X400).

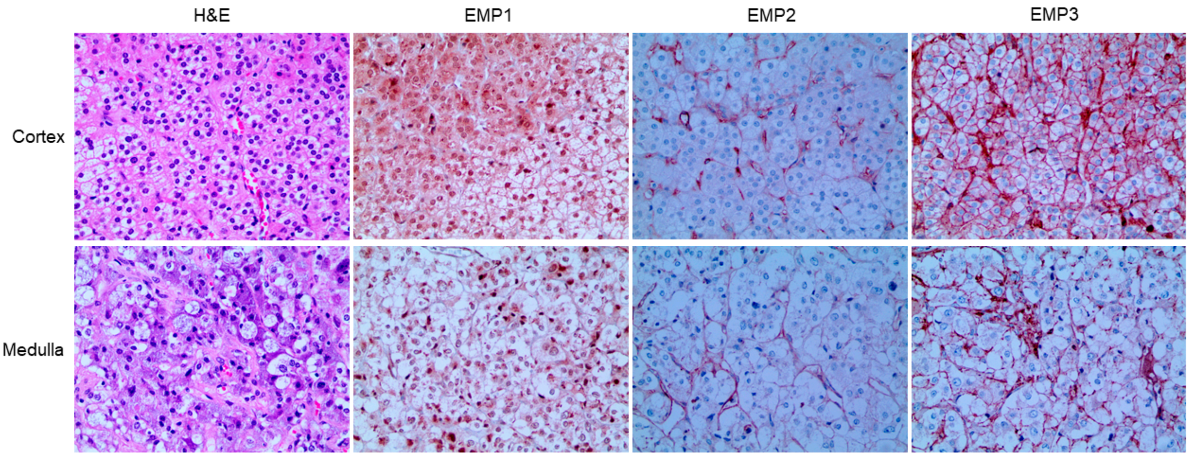

Supplement: Supplementary file 1 [file ijms-24-13016-s001.zip › ijms-2564991-supplementary.pdf]
